# Effects of RBX oleogel and heat–moisture-treated rice flour in food matrices on digestibility and microbiota

**DOI:** 10.1038/s41538-025-00418-7

**Published:** 2025-04-22

**Authors:** Tidarat Norsuwan, Thanutchaporn Kumrungsee, Noriyuki Yanaka, Tomoka Nagao, Masubon Thongngam

**Affiliations:** 1 Department of Food Science and Technology, Faculty of Agro-Industry, Kasetsart University, Bangkok, Thailand; 2 Program of Food and AgriLife Science, Graduate School of Integrated Sciences for Life, Hiroshima University, Hiroshima, Japan; 3 Smart Agriculture, Graduate School of Innovation and Practice for Smart Society, Hiroshima University, Hiroshima, Japan

**Keywords:** Dietary carbohydrates, Microbiome

## Abstract

Recently, oleogel and heat–moisture-treated (HMT) modified starch have gained much attention as a potential margarine replacement and a low-digestible starch, respectively. To date, most studies have investigated oleogel and HMT starch as individual components, while information regarding their physiological properties as a food matrix form is scarce. Here, we demonstrated that the HMT starch-oleogel food matrix exhibited the lowest plasma lipid and glucose levels, but high lipid and fecal excretion in mice, indicating that the food matrix possibly lowered lipid and carbohydrate digestibility. The resistant starch (RS) content was markedly decreased in the food matrix, suggesting other factors, such as lipid barriers and gel viscosity, in lowering the food-matrix digestibility. *Roseburia*, *Adlercreutzia*, and *rc4-4* were enriched, while *Bifidobacterium* and *Clostridium* were reduced in the food matrix group. The present study provides insights into the in vivo physiological properties and the health benefits of oleogel and HMT starch in food matrix forms.

## Introduction

Oleogelation is a technology that structures liquid oil into a semi-solid fat, termed an oleogel, by entrapping liquid oil in a fat-crystallized network^1^. Owing to their lower trans-fat content, oleogels have been proposed as a potential alternative to replace solid fats^2^. Since the development of oleogel, attempts have been made to achieve oleogel physical properties similar to those of typical shortenings. In recent years, research has shifted to understanding their physiological properties and demonstrating their use in foodstuffs. In animal studies, replacing beef tallow with oleogel in a high-fat diet (HFD) reduced lipid digestibility and increased fecal lipid excretion, thereby exerting hypolipidemic and hypocholesterolemic effects^3^, decreasing adipogenesis, and enhancing angiogenesis^4,5^. In food product applications, oleogel has been successfully applied in various foods, such as cakes, baked and steamed buns, and tortillas^6–8^. Although its physiological properties and possible applications in food products have been studied, little is known about its interactions with individual food components in the food matrix or its physiological properties in food matrix forms.

During processing, various changes occur in food components, such as carbohydrates, proteins, and lipids, leading to the formation of a food matrix via both physical and chemical interactions. Several factors, including different processing techniques and ingredients, affect the food matrix^9^. Food microstructure not only alters the textural and rheological properties of products but also affects the bioavailability of nutrients^10^. For instance, starch is a major source of carbohydrates in the human diet, and its structure changes tremendously during cooking. In recent studies, attempts have been made to modify its structure to create new textures, such as plant-based meat, or alter its digestibility to create new health functions.

Heat–moisture treatment (HMT) of starch is a physical starch modification that induces the molecular rearrangement of amylose–amylose and amylose–amylopectin interactions, as well as the formation of amylose–lipid complexes (ALCs)^11^. HMT modifies the physical and functional properties of starch, including gelatinization, pasting, and retrogradation, to improve its suitability for diverse food applications^12^. In addition, HMT can generate resistant starch (RS), which has been extensively studied for its low digestibility and prebiotic effects that confer health benefits, such as reducing postprandial glycemic responses and the risk of metabolic syndromes, including type-2 diabetes, obesity, hypertension, and heart disease, and altering microbiota composition^13^. Although RS has been greatly studied, there is a lack of information regarding the physiological properties of HMT starches or flours.

During food processing, flour is cooked, starch granules are disrupted, and interactions between starch and other food components occur, including starch–lipid interactions. Starch–lipid complexes, mostly associated with ALCs, are traditionally defined as RS type 5 (RS5)^14^. To the best of our knowledge, no in vivo studies have investigated the physiological properties of oleogels in food matrix forms, especially in HMT starch-oleogel food matrices. Most animal studies have been conducted by mixing oleogel with experimental diet-dried ingredients, including raw starch, without cooking, which does not represent the real cooking process where starch granule disruption occurs, allowing amylose–oleogel complexes to form in the food matrix. Therefore, in this study, we aimed to determine how HMT starch (in the form of flour) and oleogel in the food matrix form impact digestibility and gut microbiota.

## Results and discussion

This study aimed to investigate the in vivo physiological properties of oleogel when it is presented in food matrix forms with starch to represent the real cooking process where starch granule disruption occurs, allowing amylose–oleogel complexes to form in the food matrix. Due to an increased interest in modified starch recently, in this study, we used heat–moisture-treated flour (HMTF) as a modified starch source to determine its effects when forming a food matrix with oleogel. Rice bran wax (RBW) and rice bran oil (RBO) were used to prepare oleogel as described in our previous study^3^. Beef tallow was used as a control fat, typically used in HFD experiments. Native rice flour (NF) was used as the starch source instead of cornstarch (a typical starch used in experimental diets) and subjected to the heat moisture treatment to obtain HMTF; then, HMTF was subjected to gelatinization, which represents a cooking process, to obtain HMTF gel (referred to as HMTG). Then, we divided the experiment into six groups as shown in Table S1, which are HMTF + B, HMTG + B, HMTF + O, HMTG + O, HMTGO, and NGO. The sample preparation flow chart is shown in Fig. S1. HMTF + B, HMTG + B, HMTF + O, and HMTG + O indicated the individual component of HMTF or HMTG that was mixed with beef tallow (B) or oleogel (O) without further gelatinization or cooking process, which were used as control groups for the HMT starch-oleogel food matrix (HMTGO). HMTGO was prepared by mixing the oleogel with HMTF, followed by gelatinization, which allowed the starch in the HMTF to be gelatinized and form a food matrix with oleogel fat (HMTGO). A native starch-oleogel food matrix (referred to as NGO) was used to compare its effects to the HMTGO.

### Body weight gain, food intake, and tissue weights

As shown in Table 1, after eight weeks of feeding, the body weight gain and food intake in none of the groups differ significantly (*P* > 0.05). The liver, kidney, skeletal muscle, subcutaneous adipose tissue, and cecum content weights of all groups also did not show significant differences. The epididymal and perirenal adipose tissue weights were the highest in the HMTF + O group and lowest in the HMTG + B group. The fat tissue accumulation in the body in the HMTGO and other oleogel diet-fed groups was not different compared to the beef tallow groups. This finding is consistent with those of previous studies showing that supplementation with retrograded starch and starch–oleic acid complexes did not decrease body weight or adipose tissue weight in rodents^13,15^. Additionally, our previous study also demonstrated that oleogel replacement for beef tallow did not suppress body weight gain in rats on an HFD^3^. For the food matrix groups, HMTGO and NGO, no significant differences were observed in food intake, body weight gain, or tissue weights, including adipose tissue weights, between the two groups. This suggests that HMT in the starch-oleogel food matrix had no effects on body weight gain and fat accumulation.

**Table 1:** Body weight, food intake, tissue weights, and blood parameters

Parameters	Individual components				Food matrix	
	Beef tallow		Oleogel			
	Raw	Cooked	Raw	Cooked	Cooked	
	HMTF + B	HMTG + B	HMTF + O	HMTG + O	HMTGO	NGO
Body weight and food intake						
Initial body wt. (g)	34.0 ± 3.3	33.9 ± 2.2	34.3 ± 3.4	33.8 ± 2.2	33.9 ± 1.6	34.2 ± 2.1
Final body wt. (g)	41.0 ± 5.5	38.8 ± 2.3	42.6 ± 6.0	40.1 ± 3.7	40.3 ± 5.1	40.4 ± 6.3
Body weight gain (g/8 wks.)	6.3 ± 4.6	4.6 ± 2.1	8.3 ± 4.8	6.3 ± 3.1	6.4 ± 3.7	6.6 ± 4.5
Food intake (g/day/mouse)	3.3 ± 0.2	3.6 ± 0.3	3.4 ± 0.4	3.7 ± 0.3	4.2 ± 0.4	3.8 ± 0.6
Tissue weights						
Liver wt. (mg/g)	41.5 ± 4.0	41.5 ± 4.6	39.5 ± 3.0	40.5 ± 7.0	39.5 ± 3.8	37.4 ± 2.2
Kidney wt. (mg/g)	6.6 ± 1.0	6.3 ± 0.7	6.4 ± 0.6	6.3 ± 1.0	6.6 ± 0.7	6.5 ± 0.7
Perirenal adipose tissue wt. (mg/g)	11.0 ± 4.7^a,b^	8.0 ± 4.9^b^	15.8 ± 5.9^a^	11.6 ± 7.2^a,b^	10.5 ± 4.8^a,b^	11.5 ± 4.2^a,b^
Epididymal adipose tissue wt. (mg/g)	31.9 ± 13.5^a,b^	23.9 ± 11.4^b^	40.8 ± 13.0^a^	28.9 ± 17.0^a,b^	31.4 ± 12.8^a,b^	26.6 ± 13.3^a,b^
Subcutaneous fat wt. (mg/g)	10.0 ± 3.6	7.5 ± 1.5	11.4 ± 4.5	8.9 ± 3.7	8.6 ± 2.6	9.0 ± 5.2
Cecum wt. (mg/g)	4.7 ± 1.0	5.8 ± 0.6	5.6 ± 1.4	5.0 ± 0.7	4.4 ± 1.1	5.4 ± 1.9
Cecum content wt. (mg/g)	2.5 ± 1.0	3.6 ± 0.4	3.6 ± 1.2	3.1 ± 0.4	2.5 ± 1.1	3.2 ± 1.3
TA muscle wt. (mg/g)	1.6 ± 0.2	1.6 ± 0.2	1.5 ± 0.3	1.6 ± 0.3	1.7 ± 0.2	1.5 ± 0.1
GAS muscle wt. (mg/g)	4.6 ± 0.5	4.8 ± 0.3	4.5 ± 0.3	4.6 ± 0.4	4.9 ± 0.6	4.7 ± 0.3
SOL muscle wt. (mg/g)	0.24 ± 0.04	0.24 ± 0.02	0.23 ± 0.03	0.22 ± 0.05	0.24 ± 0.05	0.24 ± 0.05
Blood parameters related to liver function and tissue damage						
AST (IU/L)	49.8 ± 7.6	47.4 ± 6.5	47.3 ± 6.5	49.0 ± 14.6	49.9 ± 5.3	49.9 ± 10.3
ALT (IU/L)	24.1 ± 3.9	23.4 ± 4.9	24.1 ± 5.8	25.0 ± 9.1	26.1 ± 8.9	21.3 ± 3.9
T-BIL (mg/dL)	0.090 ± 0.028	0.110 ± 0.034	0.100 ± 0.023	0.095 ± 0.012	0.091 ± 0.024	0.097 ± 0.021
LDH (IU/L)	305 ± 70^b^	295 ± 56^b^	359 ± 97^a,b^	379 ± 163^a,b^	457 ± 116^a^	369 ± 107^a,b^

All values are expressed as means ± SD (*n* = 7–8 mice/group).

Different superscript letters (a and b) in the same row indicate a significantly difference by Duncan’s Multiple Range Test (*P* < 0.05).

*TA* tibialis anterior muscle, *GAS* gastrocnemius muscle, *SOL* soleus muscle, *AST* aspartate aminotransferase, *ALT* alanine aminotransferase, *T-BIL* total bilirubin, *LDH* lactate dehydrogenase.

### Dried feces and lipid profiles

**Fig. 1:**
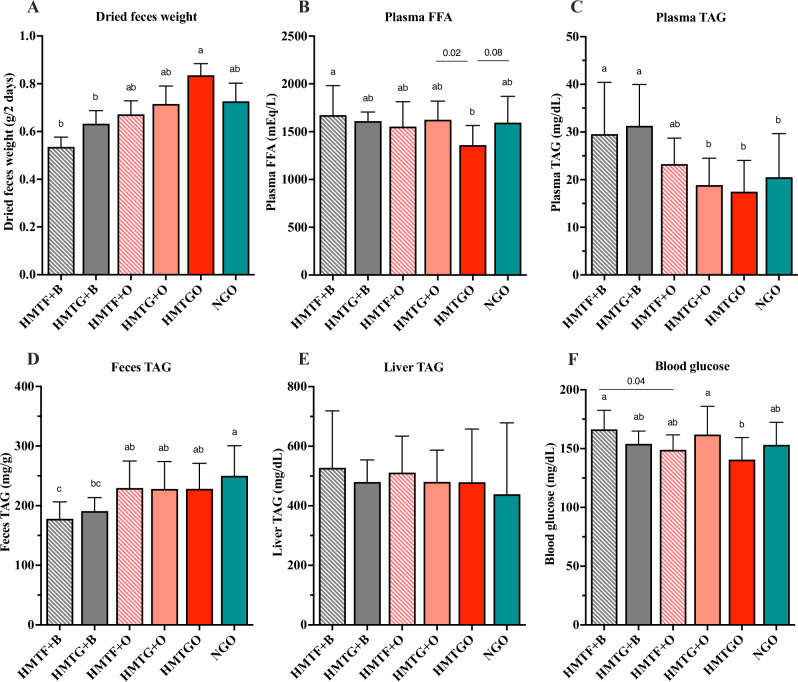
Effects of individual components and food matrices on fecal excretion, lipid profile, and blood glucose. **A** Dried fecal weight; **B** plasma FFA; **C** plasma TAG; **D** fecal TAG; **E** liver TAG; and **F** blood glucose. The results are expressed as the means ± SD (*n* = 7–8 mice/group). Different letters above the bars indicate a significant difference (*P* < 0.05). Numbers above the bars indicate *P* values analyzed using a two-tailed unpaired *t*-test with *P* < 0.05 indicating statistical significance. B beef tallow, O oleogel, HMTF heat–moisture-treated flour, HMTG HMTF gel, HMTGO HMT starch-oleogel food matrix, NGO native starch-oleogel food matrix.

For fecal excretion (Fig. 1A), the HMTGO group exhibited the highest fecal excretion, followed by the HMTG + O and NGO groups, while the HMTF + B group showed the lowest fecal excretion. The oleogel groups (HMTF + O and HMTG + O) tended to exhibit slightly higher dried fecal weights than the beef tallow groups (HMTF + B and HMTG + B). In contrast, the raw starch groups (HMTF + B and HMTF + O) exhibited slightly lower dried fecal weights than the cooked starch groups (HMTG + B and HMTG + O). Concordant with this finding, our previous study has shown that the replacement of beef tallow with oleogel in HFD increased fecal excretion^3^. These results indicate that oleogels might promote the digestibility-lowering effect, possibly increasing lipid and/or starch excretion in feces. Although no statistically significant differences were observed in dried fecal weights among the HMTGO, NGO, and HMTG + O groups, the highest fecal excretion from the HMTGO group may suggest the possibility that the digestibility-lowering effect might be attributed to the food matrix and HMT.

Next, we examined the lipid profiles and found that the HMTGO group exhibited the lowest plasma FFA levels, whereas the HMTF + B group showed the highest levels (Fig. 1B). When compared to the HMTG + O and NGO groups, the plasma FFAs in the HMTGO group were decreased by −19% (*P* = 0.02) and −17% (*P* = 0.08), respectively, suggesting that both food matrix and HMT might be the factors contributing to the plasma FFA lowering effects. The results in Fig. 1A, B indicated that the HMTGO group exhibited the highest fecal excretion and the lowest plasma FFA levels may support the hypothesis that the food matrix of HMT starch and oleogel could help lower lipid digestibility or lipid hydrolysis. Taken together with the fact that plasma ketone body levels were not significantly different among all groups (data not shown), these results imply that the starch-oleogel food matrix possibly induced suppression of dietary lipid digestion rather than promoting lipid metabolism in the body. In Fig. 1C, the HMTG + O, HMTGO, and NGO groups exhibited lower plasma TAG levels than the beef tallow groups (HMTF + B and HMTG + B), indicating that rather than food matrix or HMT, oleogel might also be the factor inducing a decrease in plasma TAG levels. In contrast, all oleogel groups showed higher fecal TAG levels than the beef tallow groups, and the HMTF + B group exhibited the lowest fecal TAG levels among all groups (Fig. 1D). This supports our hypothesis that oleogel may contribute to lowering plasma TAG levels compared to beef tallow, as observed in Fig. 1C. These results agree with those of a previous study demonstrating that oleogel replacement for beef tallow enhances lipid excretion in feces and reduces blood TAG levels without affecting body weight gain^3^. Although oleogel and its food matrix with HMT starch showed no suppressive effects on body weight or fat accumulation, their plasma lipid-lowering effects and increased fecal lipid excretion may contribute to long-term reductions in body weight and fat. Further studies are needed to confirm this hypothesis.

Taken together, it can be speculated that the food matrix and oleogel are the factors contributing to the lipid-digestibility-lowering effect, subsequently decreasing plasma lipids, and increasing lipid excretion in feces. Although changes in plasma TAG levels were observed, liver TAG levels did not differ among the groups (Fig. 1E), and fat accumulation in the liver was also not significantly different (Fig. S2).

### Blood glucose

In Fig. 1F, the HMTGO group exhibited the lowest blood glucose levels, whereas the HMTF + B group showed the highest levels. The blood glucose levels in the HMTF + O and HMTG + O groups were not significantly lower than those in the HMTF + B and HMTG + B groups; however, the blood glucose in the HMTGO group was significantly lower than that in the HMTG + O group. These results suggest that oleogel alone had less effect on blood glucose, but oleogel in the food matrix with HMT starch possibly contributed to the blood glucose-lowering effect. Moreover, the HMTGO group, followed by the NGO group, exhibited the highest fecal carbohydrate levels, which were expressed as total carbohydrate content in feces determined by a phenol–sulfuric acid method and quantified as glucose equivalent (Fig. S3). Notably, these blood glucose and fecal carbohydrate results (Figs. 1F and S3) are consistent with the plasma FFA result (Fig. 1B) and opposite with the fecal excretion result (Fig. 1A). These results suggest that the starch-oleogel food matrix promoted the lipid- and starch-digestibility lowering effect, thereby decreasing plasma lipids and glucose and increasing lipid and starch excretion in feces.

It can be speculated that the larger food volume resulting from higher food intake in HMTGO, though not significant, might have interfered with the digestion process, contributing to lower starch and lipid digestibility and higher fecal secretion. However, we hypothesized that the digestibility-lowering effect of HMTGO also plays a role. Based on our HFD Model^3^ and prior experience, CD-1 mice typically consume approximately 3–4 g of HFD per day per mouse. However, food intake fluctuates daily and can occasionally exceed 5–7 g per day. This suggests that the observed intake of 4.2 g/day in the HMTGO group did not surpass the digestive capacity of the mice. Furthermore, if HMTGO did not reduce digestibility, body weight gain should have been higher, following the general principle that greater intake leads to greater weight gain. However, despite having the highest food intake (4.2 g), the HMTGO group exhibited a body weight gain (6.4 g) similar to that of the HMTF + B group (6.3 g), which had the lowest food intake (3.3 g). Additionally, if HMTGO did not have reduced digestibility, its plasma FFA and blood glucose levels should have been higher or at least comparable to other groups, as higher food intake typically leads to greater nutrient availability. However, despite having the highest food intake, the HMTGO group exhibited the lowest blood glucose levels, while the HMTF + B group, with the lowest food intake, exhibited the highest blood glucose levels. Therefore, we hypothesize that the digestion process is affected not by the large consumed volume of HMTGO but rather by its food matrix, which reduces starch and lipid digestibility and increases fecal secretion.

To date, several studies have incorporated oleogels into experimental diets without cooking, which prevents the formation of starch–lipid complexes or food matrices; thereby, the effects of oleogel in food matrices on the physiological functions of food have remained elusive. Issara and Park^4^ has shown that feeding an HFD containing beef tallow with cornstarch (raw) to rats for 5 weeks increased blood glucose to 165 mg/dL in the HFD control group (equivalent to the HMTF + B group in the present study), and the replacement of oleogel for beef tallow decreased blood glucose to 135 mg/dL (equivalent to the HMTF + O group in the present study). Consistent with these findings, our results revealed decreased blood glucose levels in the HMTF + O group compared to those in the HMTF + B group (148.9 ± 12.8 vs 166.3 ± 16.3, *P* = 0.04, Fig. 1F). In other related studies, feeding retrograded starch or starch–oleic acid complexes to HFD-fed rodents exhibited an opposite result of increasing blood glucose levels^12,13^. To the best of our knowledge, the present study is the first to demonstrate in vivo effects of oleogel in food matrix forms.

### Cholesterol profiles and liver function

**Fig. 2:**
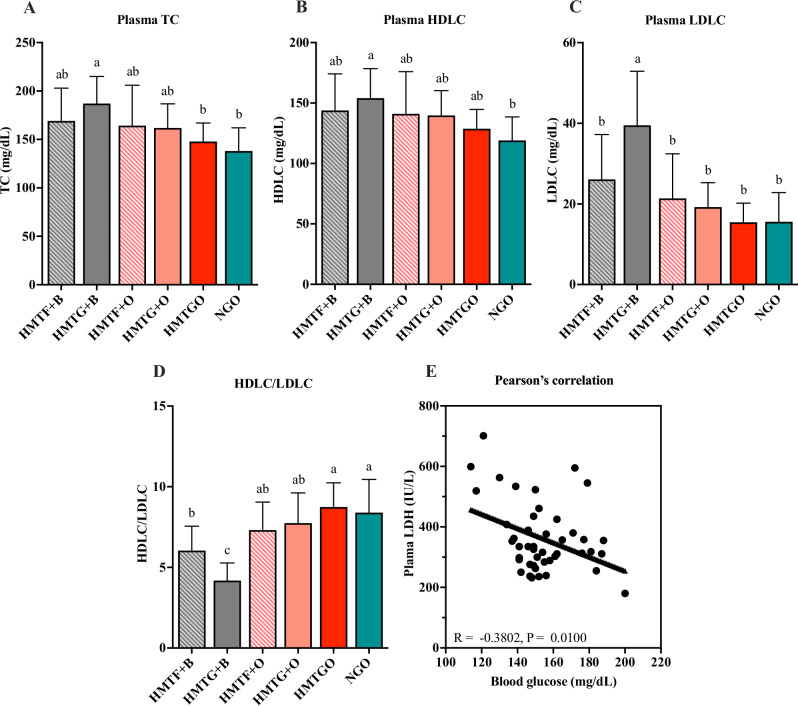
Effects of individual components and food matrices on plasma cholesterol profile and a correlation between plasma lactate dehydrogenase and blood glucose. **A** Plasma TC; **B** plasma HDLC; **C** plasma LDLC; **D** HDLC/LDLC ratio, and **E** Pearson’s correlation between plasma LDH and blood glucose. The results are expressed as the means ± SD (*n* = 7–8 mice/group). Different letters above the bars indicate a significant difference (*P* < 0.05). B beef tallow, O oleogel, HMTF heat-–moisture-treated flour, HMTG HMTF gel, HMTGO HMT starch-oleogel food matrix, NGO native starch-oleogel food matrix, TC total cholesterol, HDLC high-density lipoprotein cholesterol, LDLC low-density lipoprotein cholesterol, LDH lactate dehydrogenase.

As shown in Fig. 2A–C, the food matrix groups (HMTGO and NGO) showed the lowest levels of TC, LDLC, and HDLC, whereas the beef tallow groups (HMTF + B and HMTG + B) showed the highest trends in these parameters. Furthermore, TC, LDLC, and HDLC levels were slightly lower in the oleogel groups than those in the beef tallow groups. In contrast, the HDLC/LDLC ratios were significantly higher in the HMTGO and NGO groups and slightly higher in the HMTF + O and HMTG + O groups than those in the HMTF + B and HMTG + B groups (Fig. 2D). These results suggest that oleogel could lower blood cholesterol and increase the HDLC/LDLC ratio compared to beef tallow. Cholesterol is a product of lipid digestion^16^; therefore, the low levels of TC might be attributed to the low lipid digestion of oleogel (Fig. 1C, D).

For blood parameters related to liver function, the AST, ALT, and T-BIL levels in all groups did not differ significantly (Table 1), indicating no difference in liver health. However, plasma LDH level was the highest in the HMTGO group and the lowest in the HMTF + B and HMTG + B groups. LDH, ALT, and AST are present in various body tissues, including skeletal muscle, liver, kidney, heart muscle, pancreas, and brain^17^ and are considered the markers of tissue damage or injury because they are released from damaged cells into the bloodstream. In the present study, AST and ALT levels did not differ between the tested groups. In addition, skeletal muscle, kidney, and liver tissue weights did not differ between groups (Table 1). These results suggest that the high LDH levels in the HMTGO group may indicate factors other than tissue damage. Besides serving as a tissue damage marker, LDH also plays a crucial role in glycolysis and gluconeogenesis (or glucose production). In glycolysis, glucose is changed to pyruvate, then pyruvate is converted to lactate by LDH (the A isoform, LDHA); reversibly, gluconeogenesis is a process that transforms lactate back into glucose, through the processes of converting lactate to pyruvate by LDH (the B isoform, LDHB) and converting pyruvate to glucose^18^ (Fig. S4). As shown in Table 1 and Fig. 2E, the HMTGO group showed the highest plasma LDH and the lowest blood glucose levels, whereas they were opposite in the HMTF + B group. This result suggests that LDH might be negatively correlated with blood glucose, which was confirmed by a moderately negative correlation (*r* = −0.3802, *P* = 0.010; Fig. 2E) between plasma LDH and blood glucose levels as assessed using Pearson’s correlation coefficient analysis. The lower blood glucose level in the HMTGO group may feedback-upregulate the activity of LDH (LDHB) in the gluconeogenesis pathway in the liver to release more glucose into the bloodstream to compensate for low blood glucose, preventing hypoglycemia (Fig. S4). In contrast, high blood glucose levels (as in the HMTF + B group) may blunt or downregulate LDH (LDHB) activity (Fig. S4). Our hypothesis is supported by the finding that increased glucose availability in the brain by glucose infusion reduces glucose production in neurons, where lactate is the main substrate for glucose production, and LDH is a critical regulator of glucose levels and glucose production^19,20^. Another study showed that mice fed an HFD exhibited high blood glucose with a lower activity of LDHB, responsible for converting lactate to pyruvate, but had no effects on the activity of LDHA^18^. Studies have shown that decreased LDH activity impairs lactate clearance in the liver, causing lactate accumulation that provokes lipid accumulation and an inflammatory response^18^. Together, our findings suggest the possibility that the starch-oleogel food matrix improves glucose and energy metabolism and exerts hepatoprotective effects. In the future, to prove this glucose-LDH hypothesis, further direct studies on the hepatic LDHB activity are necessary.

### RS content

It has been hypothesized that HMT generates RS, and cooking or gelatinization induces the interactions between starch and lipid to form starch–lipid complexes that are defined as RS5^14^. Therefore, we have expected that enhancing RS due to HMT and the starch–lipid (oleogel) complexes would possibly contribute to the digestibility-lowering effects shown in Fig. 1. Then, we measured RS content in samples and found that the heat moisture treatment significantly increased the RS content in flour (1.59 ± 0.19 (NF) vs 4.10 ± 0.05 (HMTF) g/100 g, *P* < 0.05, Table S2). This result is in agreement with our recent study^21^ and other studies demonstrating that HMT starch has high RS content^22,23^ and HMT promotes the formation of ALCs or RS5^24,25^. Unexpectedly, cooking or gelatinization of HMTF did not increase the RS content (4.10 ± 0.05 (HMTF) vs 3.63 ± 0.01 (HMTG) g/100 g, *P* < 0.05, Table S2), and incorporation of oleogel during the gelatinization of HMTF to form the starch-oleogel matrix further decreased the RS content (3.63 ± 0.01 (HMTG) vs 2.69 ± 0.01 (HMTGO_10 (10% oleogel)), or 1.93 ± 0.01 (HMTGO_20 (20% oleogel)) g/100 g, *P* < 0.05, Table S2). As compared to HMTF, whose starch remained in a granular structure, we hypothesized that starch granules in HMTG were ruptured during gelatinization, which facilitated and increased the accessibility of amylase to its substrates, amylose and amylopectin, thereby reducing the RS content in HMTG. In line with our hypothesis, a study demonstrated that the RS contents of gelatinized HMT rice flours were markedly lower than those of ungelatinized HMT rice flours (decreased by more than 60%)^26^. Another study demonstrated that the RS content of native canna starch was greatly decreased when the native starch was subjected to gelatinization (decreased by more than 80%)^27^. Li et al.^26^ proposed that the lower RS contents in gelatinized HMT rice flours when compared to ungelatinized HMT rice flours might be attributed to an irreversible collapse of the starch granules and crystalline structures due to gelatinization.

As shown in Table S2, the RS content of HMTGO decreased as the concentration of oleogel increased. To date, there are only a few studies applying oleogel in food matrices, and the effects of oleogel supplementation on RS contents and digestibility are controversial. A study showed that substituting butter in 0–100% by candelilla wax/canola oil oleogel (0–9% oleogel in cake batter) increased crystalline structures of starch chains, the structure resistant to hydrolysis, reduced the formation of amylose–lipid complexes, but increased in vitro starch digestibility in cake as the oleogel content was increased^7^. The mechanisms underlying increased digestibility due to candelilla wax/canola oil oleogel supplementation in this study remain unclear^7^. In contrast, incorporation of candelilla wax/canola oil oleogel (2–6%) in masa used for tortillas significantly increased the RS content, increased the formation of starch–lipid complexes, and decreased in vitro starch digestibility in tortillas as the concentration of oleogel increased^6^. The team proposed that a great decrease in rapidly digestible starch (RSD) fractions (−46%) due to the oleogel incorporation could not solely resulted from the formation of ALCs, but rather the formation of an oleogel oily layer around the starch granules that in turn isolated the starch chains and granules, thereby reducing the accessibility of amylolytic enzymes to the starch chains resulting in a decreased in starch digestibility^6^. Replacement of margarine with sunflower or olive oil oleogel (10%) was found to have no effects on in vitro lipid digestibility in steamed and baked buns^8^.

In our present study, the concentration of oleogel (30%) in HMTGO used in the HFD formulation is higher than those oleogel concentrations (2–10%) used in cake, tortillas, and buns. It can be postulated that the high oleogel concentration (30%) might greatly form an oily barrier surrounding the starch chains and granules, hindering the access of enzymes from the digestive juice to the starch chains subsequent to a decrease in starch digestibility as proposed by Vernon-Carter et al.^6^. In a high lipid content environment, lipids might self-assemble to form lipid-lipid interactions rather than to form starch–lipid interactions during cooking or gelatinization^28^, thereby possibly reducing the RS content in HMTGO. Moreover, a high lipid content environment can increase the overall or final viscosity of a mixture (Table S3). When gelatinized starch is present in such a high-viscosity environment, its mobility can be limited. The starch molecules may become more entangled in the lipid-rich matrix, hindering their movement. This limitation of gelatinized-amorphous matrix mobility could reduce starch digestibility^29^. Furthermore, the viscosity of gels can limit the diffusion rate of digestive enzymes, slowing down the hydrolysis^30^. Although the RS content was decreased in HMTGO, the digestibility (of both starch and lipid) was decreased, reflected in the increase of fecal secretion and fecal carbohydrate content and the decrease of blood lipid and glucose levels (Figs. 1 and S3). The result suggests that the RS content may not be the sole primary factor contributing to decreased digestibility in HMTGO. We proposed that other factors, such as lipid barriers and gel viscosity, in the HMTGO may also play a role in decreasing digestibility in HMTGO.

As shown in Fig. S5, we conducted a preliminary experiment to observe the morphology of cooked HMTG and HMTGO using scanning electron microscopy (SEM). In Fig. S5A, without lipid (oleogel) addition, the SEM image revealed a porous structure, likely indicating a fully gelatinized starch structure in HMTG. Conversely, incorporating oleogel into HMTG led to the formation of starch–oleogel complexes in HMTGO, producing a more heterogeneous structure compared to HMTG (Fig. S5B). The HMTGO structure (Fig. S5B) exhibited round-surface structures (yellow circles), which were absent in HMTG (Fig. S5A). We speculate that these features may represent lipid (oleogel) integrated into the complex matrix. A previous study^31^ also demonstrated that RBO-based oleogel exhibited round-surface structures, analyzed by SEM, similar to those observed in our study. The inclusion of this lipid (oleogel) in the complex structure might hinder the accessibility of digestive enzymes, potentially reducing the digestion as discussed above. To further demonstrate the potential inclusion of lipid (oleogel) in the HMTGO structure, we employed Fourier transform infrared (FTIR) spectroscopy. As shown in Fig. S6, the FTIR spectra of both HMTGO samples (10% and 30% oleogel, Fig. S6B, C) exhibited absorbance peaks at 2921–2920 cm^−1^, 2851–2850 cm^−1^, and 1744 cm^−1^, corresponding to the methyl (CH₃), methylene (CH₂), and ester groups of fatty acids and TAG in lipid^32,33^, while these peaks were absent in HMTG (Fig. S6A), indicating the presence of oleogel in the HMTGO structure. Previous studies have identified peaks at 1744–1743 cm^−1^ as the ester carbonyl group, potentially formed by esterification between lipid and starch, as observed in starch–lipid complexes from maize starch-oil mixtures^34^ and maize tortillas^35^. Additionally, research has shown that the peak intensity at 1743 cm^−1^ in the maize starch-soybean oil–water food matrix increased with higher moisture levels used to hydrate starch before cooking^32^. At high moisture levels, starch granules swell and partially gelatinize, facilitating lipid absorption into both external and internal fractions of starch granules, thereby enhancing starch–lipid complex formation, as reflected by the increased peak intensity at 1743 cm^−1^ ^32^. In our study, we observed that the peak intensity at 1744 cm^−1^ likely increased with higher oleogel incorporation in the HMTGO food matrix (Fig. S6B, C), suggesting enhanced starch–lipid interactions in HMTGO with 30% oleogel. Taken together, we hypothesized that the 1744 cm^−1^ peaks in HMTGO indicate starch-oleogel interactions within the food matrix. Future studies should further elucidate the more specific interactions or binding mechanisms between oleogel and HMT starch within the food matrix. These will reveal more morphological structures of starch–oleogel complexes, such as oleogel oily barriers or layers, that are hypothesized to influence the physical properties of HMTGO, including gel viscosity and digestibility.

### Gut microbiota composition

**Fig. 3:**
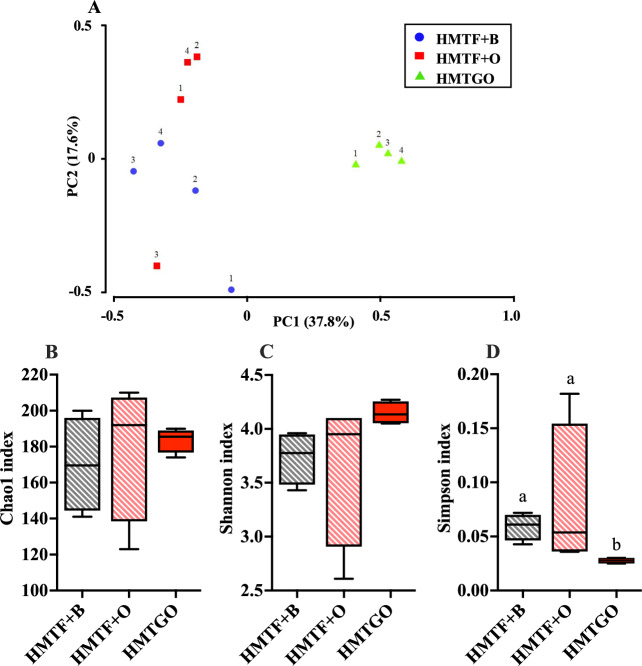
Effects of the HMTGO and its control individual components and uncooked groups on gut microbiota composition. **A** Principal component analysis (PCA) of the ASV—level Bray–Curtis distance and alpha diversity analysis; **B** Chao1 index; **C** Shannon index, and **D** Simpson index. Data are presented as a boxplot with median and min–max whiskers (*n* = 4). Different letters above the bars indicate a significant difference (*P* < 0.05). B beef tallow, O oleogel, HMTF heat–moisture-treated flour, HMTGO HMT starch-oleogel food matrix, NGO native starch-oleogel food matrix.

Effects of the starch-oleogel food matrix on the gut microbiota composition of the HMTGO group were compared with those of its control uncooked groups, HMTF + B and HMTF + O. Principal coordinate analysis (PCoA) revealed that the microbial structures of the HMTF + B and HMTF + O groups slightly overlapped, suggesting a similarity of the microbial community structures between the two groups (Fig. 3A). In contrast, the HMTGO group was distinctly separated from the HMTF + B and HMTF + O groups, indicating that oleogel in the food matrix form with starch significantly altered the gut microbiota composition (Fig. 3A). As shown in Fig. 3B, there are no significant differences in the Chao 1 index among the three groups, indicating a similar richness of gut bacteria among the groups. In addition, the Shannon index of the HMTGO group was slightly higher, but the Simpson index was significantly lower than those of the HMTF + B and HMTF + O groups, suggesting that the starch-oleogel food matrix enhanced species diversity, whereas the change in fat sources (beef tallow or oleogel) did not exhibit such an effect under the uncooked conditions (Fig.3C, D).

**Fig. 4:**
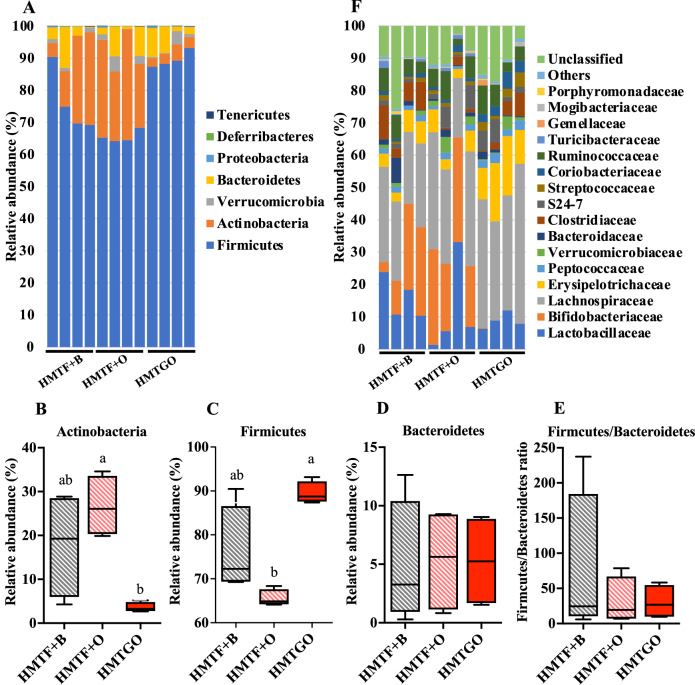
Effects of the HMTGO and its control individual components and uncooked groups on gut microbiota composition. At phylum (**A**) and family (**F**) levels. **B** *Actinobacteria* phylum; **C** Firmicutes phylum; **D** Bacteroidetes phylum; and **E** Firmicutes/Bacteroidetes ratio. Data are presented as a boxplot with median and min–max whiskers (*n* = 4). Different letters above the bars indicate a significant difference (*P* < 0.05). B beef tallow, O oleogel, HMTF heat–moisture-treated flour, HMTGO HMT starch-oleogel food matrix, NGO native starch-oleogel food matrix.

**Fig. 5:**
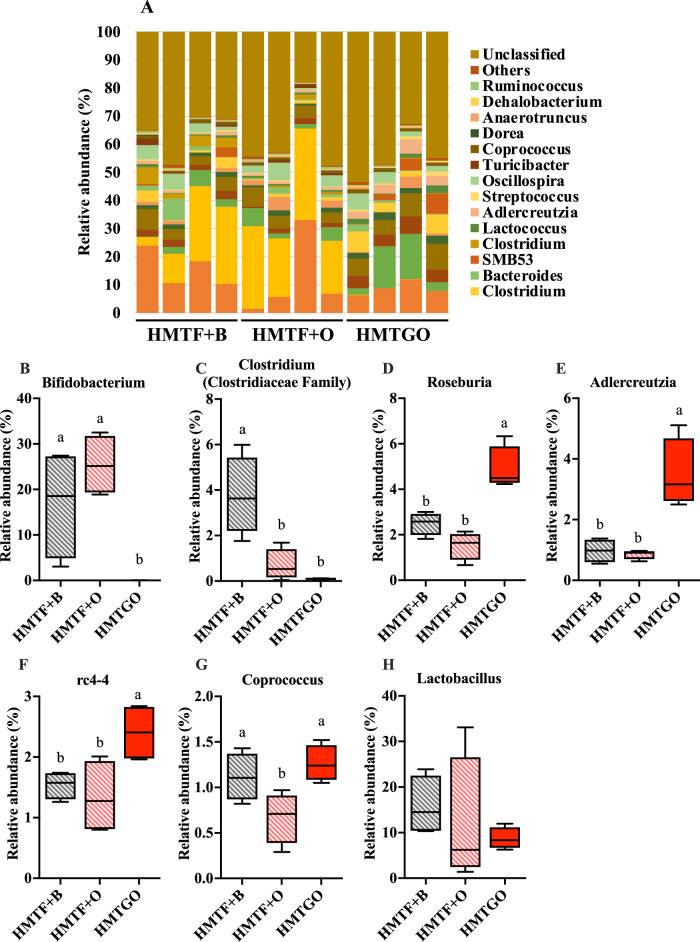
Effects of the HMTGO and its control individual components and uncooked groups on gut microbiota composition at genus levels. Gut microbiota composition at genus levels (**A**). **B**–**G** Significantly changed genera among the treatments: **B** *Bifidobacterium*; **C** *Clostridium*; **D** *Roseburia*; **E** *Adlercreutzia*; **F** rc4-4; **G** *Coprococcus*; and **H** *Lactobaciullus*. Data are presented as a boxplot with median and min–max whiskers (*n* = 4). Different letters above the bars indicate a significant difference (*P* < 0.05). B beef tallow, O oleogel, HMTF heat–moisture-treated flour, HMTGO HMT starch-oleogel food matrix, NGO native starch-oleogel food matrix.

As shown in Fig. 4A, among the seven most abundant phyla, the HMTGO group exhibited a significant decrease in the relative abundance of *Actinobacteria* (Fig. 4B), but a significant increase in that of *Firmicutes* (Fig. 4C), as compared to the HMTF + B and HMTF + O groups. However, the abundance of *Bacteroidetes* and the ratio of *Firmicutes*/*Bacteroidetes* were not different between the three groups (Fig. 4D, E). The top 16 bacterial taxa at the family level are shown in Fig. 4F. Compared to the HMTF + B and HMTF + O groups, the HMTGO group showed a significantly increased relative abundance of *Coriobacteriaceae*, *Erysipelotrichaceae*, and *Peptostreptococcaceae*, but a significantly decreased abundance of *Bifidobacteriaceae* with no effect on *Lactobacillaceae*. The relative abundance of *Clostridiaceae* was significantly lower in the HMTF + O group than that in the HMTF + B and HMTGO groups (Fig. 4F). At the genus level (Fig. 5A–H), a total of 6 genera showed significant changes (*P* < 0.05) between the three groups. When compared to the HMTF + B and HMTF + O groups, the relative abundance of *Bifidobacterium* was significantly decreased (Fig. 5B), while the relative abundance of *Roseburia*, *Adlercreutzia*, and *rc4-4* was significantly increased (Fig. 5D–F) in the HMTGO group. For *Coprococcus*, its relative abundance was significantly decreased in the HMTF + O group (Fig. 5G), while the relative abundance of *Clostridium* was significantly higher in the HMTF + B group (Fig. 5C). The relative abundance of the well-known probiotic *Lactobacillus* was not changed across the groups (Fig. 5H).

To date, there is no study demonstrating the effects of oleogel either as an individual food component (equivalent to the HMTF + O group in the present study) or as in food matrices (equivalent to the HMTGO group in the present study) on gut microbiota. Our study is the first to provide evidence that oleogel in the food matrix form (HMTGO) impacted gut microbiota differently from oleogel itself alone (HMTF + O). Oleogel itself alone as an individual food component (HMTF + O) had fewer effects on changing gut microbiota composition, reflected by similar changes in gut microbiota in the HMTF + B group (Fig. 5A–H).

Interestingly, in the present study, the starch-oleogel food matrix (HMTGO) markedly decreased the abundance of *Bifidobacterium*, which is against our expectation that a decrease in digestibility of the starch-oleogel food matrix might possibly support the growth of probiotics. In a previous study, it was demonstrated that a rice starch–oleic acid complex supplementation in HFD (15% lipid) increased the abundance of *Bifidobacterium* but decreased that of the probiotic *Lactobacillus*^13^. Another study showed that a retrograded starch supplementation in HFD (24% lipid) did not affect these two probiotics^15^. Wang and coworkers^36^ and Bendiks and coworkers^37^ have suggested that the adhesion of *Bifidobacterium* to starch granules is a crucial function that promotes bacterial growth and survival. *Bifidobacterium pseudolongum* (a predominant *Bifidobacterium* species identified in the present study) is a highly starch-granule adherent species, and treatment with pancreatin, inducing starch granule hydrolysis, induces a loss of adhesion of *Bifidobacterium* to the starch by 50%^38,39^. These findings may explain why the abundance of *Bifidobacterium* decreased in the HMTGO group compared to that in the HMTF + B and HMTF + O groups. In the HMTGO group, gelatinization during the cooking process disrupted starch granules, possibly induced networking of the oleogel with amylose, or induced the oleogel oily barrier isolating the starch chains and granules, which further hindered starch granules or amylose from *Bifidobacterium* adhesion. In contrast, in the HMTF + B and HMTF + O groups, there was no cooking process; thus, the starch remained intact in granular form, which possibly enhanced the starch adhesion of *Bifidobacterium*, thereby promoting the growth and survival of the bacteria. In addition, recent research has demonstrated that the order *Coriobacteriales*, which shares the same phylum of *Actinobacteria* as the order *Bifidobacteriales*, also produces phosphoketolases, *Bifidobacterium*-specific enzymes, that hydrolyze fructose-6-phosphate and xylulose-5-phosphate substrates^40^. This suggests that *Adlercreutzia* (Fig. 5E) of the *Coriobacteriaceae* family (Fig. 4F), which was highly increased in the HMTGO group, may compete with *Bifidobacterium* for their substrates, thereby suppressing the growth of *Bifidobacterium*. However, the relative abundance of *Adlercreutzia* (about 4%) was far lower than that of *Bifidobacterium* (about 30%) (Fig. 5E, B). Further studies are highly required to reveal the impact of oleogel on gut microbiota.

The abundance of *Roseburia* was increased in the HMTGO group. With its butyrate-producing properties, *Roseburia* has recently been proposed as a new potent probiotic^41^ with various health benefits, such as the regulation of energy metabolism, the gut barrier, immune function, and atherosclerosis^42^. Another butyrate-producing genus, *Coprococcus*, has also been proposed to be a potent probiotic, recently^43^. *Coprococcus* was reported to be able to grow well on various prebiotics, such as β-glucans, galactomannan, galactan, glucomannan, and starch^44^. Its abundance is inversely correlated with several diseases, such as neurodegenerative disorders, a systemic autoimmune disorder, and inflammatory bowel disease^43,45,46^. For *Adlercreutzia* and *rc4-4*, the information regarding their responses to prebiotics and health benefits is very limited. Although HMTGO markedly decreased the abundance of the probiotic *Bifidobacterium*, it also markedly decreased the well-known human pathogen genus *Clostridium*.

In this microbiota analysis, there are some limitations. First, only three groups—HMTGO, HMTF + B, and HMTF + O—were analyzed. Second, we used pooled cecal samples (two mouse cecal samples per pooled sample). To more fully clarify the effects of oleogel, cooking process (gelatinization), HMT, and the starch-oleogel food matrix on gut microbiota, microbiota analysis of additional groups will be needed in the future. Furthermore, individual cecal analysis of each mouse will provide more precise insights into the effects of oleogel and its food matrix on gut microbiota. Although pooling cecal samples is not an ideal practice, some studies have also conducted microbiota analysis using pooled samples^47,48^. Given the lack of available studies on the effects of oleogel on gut microbiota, we believe that the microbiota results from our current study could provide valuable new insights into the field.

As discussed above, it appears that lipid content in food matrices affects probiotics, such as *Bifidobacterium*. Lower lipid content in food matrices (15% or 24%) either increased or had no effect on the abundance of *Bifidobacterium* or *Lactobacillus*^13,15^, whereas higher lipid content (30% lipid (oleogel)) significantly reduced the abundance of *Bifidobacterium* (Fig. 5B). Additionally, the high lipid content (30%) also showed potential to reduce RS content (Table S2). These findings highlight the importance of evaluating multiple factors, such as oleogel content, when applying oleogel in food processing to produce health-beneficial, oleogel-based food products.

In summary, we demonstrated that oleogel in the food matrix with HMT starch (in the form of flour) (HMTGO) exhibited the in vivo physiological properties differently from oleogel itself alone (HMTF + O). The HMTGO exhibited the highest fecal excretion and improved blood parameters, the lowest blood FFA, TAG, and glucose levels, and the highest HDLC/LDLC ratio. The RS content was markedly decreased due to gelatinization and oleogel incorporation; we proposed that lower digestibility of HMTGO might be attributed to other factors beyond the RS content, such as lipid barriers and gel viscosity. In terms of effects on gut bacteria, oleogel in the food matrix form (HMTGO) impacted gut microbiota differently from its control, uncooked, and oleogel alone group (HMTF + O). The HMTGO increased the abundance of *Roseburia*, *Adlercreutzia*, and *rc4-4* genera, whereas it decreased the abundance of *Bifidobacterium* and *Clostridium* genera. The abundance of *Coprococcus* was reduced in the HMTF + O group. The present study provides insights into the in vivo physiological properties of oleogel in the food matrix form with HMT starch and its potential health benefits and implementation in food processing.

## Methods

### Materials

RBW (melting point: 77–83 °C, acid value: ≤10, saponification value: 70–95, iodine value: ≤13, ester content: 94%, free fatty acid: 4%, free fatty alcohol: 1%, hydrocarbon: 1% and others: 12 ppm) was purchased from Yokozeki Oil & Fat Industries Co., Ltd. (Tokyo, Japan). RBO containing oryzanol (8000 ppm), phytosterols (12,000 ppm), and vitamin E (tocopherols and tocotrienols) was obtained from Thai Edible Oil Co., Ltd. (Samutprakarn, Thailand). Beef tallow was purchased from Nacalai Tesque, Inc. (Kyoto, Japan). Polished rice grains (Chainat 1) with high amylose content (32.3% amylose, 6.2% protein, and 88.0% total starch, % *w*/*w* in flour) were obtained from the Rice Department (Bangkok, Thailand).

### RBW–RBO oleogel preparation

An oleogel containing 7.5% (*w*/*w*) RBW and 3.75% (*w*/*w*) glycerol monostearate (GMS) was prepared by dissolving RBW and GMS in RBO. The GMS–RBW–RBO mixture was heated to 90 °C with constant stirring to completely melt RBW and GMS crystals. Then, the mixture was cooled to 20 °C by a circulating water bath to solidify the mixture, allowing oleogel to form. The RBW–RBO oleogel was kept at 4 °C overnight to strengthen the oleogel structures and then kept at 4 °C or room temperature until further use.

### Native and heat–moisture-treated rice flour preparation

The polished rice grains were wet milled using a colloidal mill. Then the extra water was removed from the rice slurry by centrifugation and dried in a tray dryer at 45 °C until 10–12% moisture content was reached. Then the dried slurry was ground using a rotor mill and passed through a 100-mesh sieve (150 µm) to obtain the rice flour (NF), which was used in the subsequent analyses. NF was packed in a polyethylene bag, sealed, and stored at −18 °C.

To prepare HMTF, NF (100 g, dry weight) was weighed and placed in an aluminum foil pouch. Then, distilled water was added to reach a moisture content of 20% *w*/*w*, followed by equilibration of the moistened flour samples at 4 °C for 24 h. The sample was then heated at 110 °C for 14 h and dried at 45 °C until the moisture content reached approximately 9–10%. Then, the samples were cooled, ground, passed through a 100-mesh sieve, and kept in a polyethylene bag at −18 °C^49^.

### Heat–moisture-treated rice flour gel preparation

HMTF was mixed with water (20% *w*/*w*) to form an HMTF suspension. Then the suspension was stirred at 25 °C at 750 rpm for 10 min, then heated to 68 °C over 12 min using a hot plate stirrer. The HMTF paste was poured onto the aluminum trays and steamed at approximately 100 °C for 20 min before cooling to 25 °C for 3 h. Subsequently, the HMTF gel (HMTG) was dried in a hot air oven at 55 °C for 10 h (the final water content of the gel was maintained at 40–45% *w*/*w*) and stored at 4 °C until further use.

### Starch-oleogel food matrix preparation

RBW–RBO oleogel (30% *w*/*w*) was added to the NF or HMTF suspension (20% w/w) &&and stirred at 25 °C at 750 rpm for 10 min. Afterward, the suspensions were heated to and stirred on a hot plate stirrer for 12 min until the paste temperature reached 68 °C. The pastes were poured onto the aluminum trays, steamed at approximately 100 °C for 20 min, and cooled to 25 °C for 3 h. In this step, the starches in NF and HMTF were gelatinized and networked with lipids in the oleogel, forming the NF or HMTF starch-oleogel food matrix (NGO or HMTGO, respectively). NGO and HMTGO were dried in a hot air oven at 55 °C for 10 h and kept at 4 °C until further use.

### Animals and experimental design

Seven-week-old male CD-1 (ICR) mice were purchased from Charles River Japan (Hino, Japan) and maintained according to the “Guide for the Care and Use of Laboratory Animals” established by Hiroshima University and approved by the Ethics Committee of Hiroshima University (ethical approval no. C22-31-2). The mice were housed individually in stainless–steel cages in a windowless, air-conditioned room (24 ± 1 °C) with 50 ± 10% relative humidity under a 12 h light-dark cycle. The mice had free access to food and drinking water and were acclimatized to a non-purified commercial rodent diet (MF; Oriental Yeast Co., Ltd., Tokyo, Japan) for 7 days before starting the experimental diet.

Mice were randomly divided into six groups (*n* = 8/group) and fed six different HFDs, as shown in Table S1. The HFD comprised (g/kg diet):-cornstarch, 202; casein, 200; sucrose, 200; beef tallow, 300; cellulose, 50; AIN-93G mineral mixture, 35; AIN-93 vitamin mixture, 10; and l-cysteine, 3, as previously described^3^. Cornstarch, as a control carbohydrate source, was replaced by raw HMT starch (HMTF) or cooked HMT starch (HMTG), whereas beef tallow (control fat source) was replaced with the RBW–RBO oleogel. In Table S1, the first four HFDs (HMTF + B, HMTG + B, HMTF + O, and HMTG + O) were prepared by mixing the dried ingredients, adding flour or gel (HMTF or HMTG) and fat [beef tallow (B) or RBW-RBO oleogel (O)] without melting, and manually mixing until the diets were homogenous. These four HFDs served as the control for the HMTGO group to determine the individual component effects. The final two HFDs were prepared by mixing the oleogel with the NF or HMTF before heating and cooling to form a gel (NGO or HMTGO). The HMTGO group served as the treatment group for determining the effects of the HMTGO, whereas the NGO group was used to compare the effects of the HMT and native flours. The diets were prepared every week and stored at 4 °C. All six experimental diet formulations were adjusted to have similar protein, lipid, and carbohydrate compositions. Food intake and body weight were measured twice weekly throughout the feeding period of eight weeks. The fecal samples were collected weekly.

At the end of the feeding period, all mice were fasted for 6 h before being sacrificed by cervical dislocation under isoflurane anesthesia (between 13:00 and 15:00). Blood was collected at dissection from the abdominal vein in tubes containing heparin (an anticoagulant) on ice. Then, plasma was obtained by centrifugation at 3500 rpm for 20 min and stored at −80 °C. The liver, kidney, fats (perirenal, epididymal, and subcutaneous fats), cecum, cecum content, and muscles (tibialis anterior; TA, gastrocnemius; GAS, and soleus; SOL muscles) were removed immediately, weighed, and stored at −80 °C until analysis. The percentage of tissue weight was calculated as tissue weight (mg) per final body weight (g)^3^.

### Blood parameter analysis

Blood parameters were chosen mainly to assess the lipid profile, glucose levels, liver function, and tissue damage. Triacylglycerol (TAG), total cholesterol (TC), free fatty acids (FFAs), high-density lipoprotein cholesterol (HDLC), and low-density lipoprotein cholesterol (LDLC) were used as lipid parameters, while aspartate aminotransferase (AST), alanine aminotransferase (ALT), gamma-glutamyltransferase (γ-GT), and total bilirubin were used as liver function parameters. Lactate dehydrogenase (LDH) was used as a marker of tissue damage. These biochemical indicators in plasma were determined using previously described methods^3^. TAG and TC concentrations were quantified using commercial enzymatic kits (Wako Pure Chemicals, Osaka, Japan). HDLC, LDLC, FFA, glucose, AST, ALT, LDH, and total bilirubin levels were determined using a Beckman Coulter AU480 analyzer (Beckman Coulter, Krefeld, Germany), which is an automated chemistry instrument for turbidimetric, spectrophotometric, and ion-selective electrode measurements. Briefly, 200 μL plasma was used to measure these parameters according to the manufacturer’s protocol. Blood glucose (6 h fasting) was measured using a glucose meter (ACCU-CHECK, Roche, Tokyo, Japan).

### Hepatic lipid analysis

Liver lipids were extracted using Folch’s method (1957)^50^ with some modifications. Fresh liver (500 mg) was minced and homogenized in 2.0 mL of 0.14 M KCl solution for 2 min. Then, 0.5 mL of homogenized solution was added to 9.5 mL of chloroform/methanol (2:1, *v*/*v*) and shaken at 4 °C overnight before filtering. The filtrate (3.5 mL) was mixed with 0.75 mL 0.02% CaCl_2_ solution and centrifuged at 2000 × *g* for 10 min. The mixture was separated into two phases (upper and lower phases), and the lower phase was collected and mixed with 0.75 mL chloroform: methanol: 0.02% CaCl_2_ (3:48: 47, *v*/*v*). After centrifuging the mixture at 2000 × *g* for 10 min, the lower phase was collected, diluted with 1.0 mL methanol, 2.0 mL chloroform: methanol (2:1, *v*/*v*) was added before evaporating at 60 °C. The samples were dissolved in 270 µL tert-butanol and 180 µL Triton X-100: methanol (1:1, *v*/*v*) before quantifying the hepatic TAG concentration using commercial enzymatic kits (Wako Pure Chemicals, Osaka, Japan)^3^.

### Fecal lipid analysis

The collected wet feces were freeze-dried for 24 h and ground. Dried feces (0.250 g each) were used to extract lipids using the above-mentioned method. The fecal TAG concentrations were measured using commercial enzymatic kits (Wako Pure Chemicals, Osaka, Japan).

### Histological staining

A small piece of liver was fixed in 10% buffered formalin. The liver tissues were then placed in embedding cassettes for dehydration using ethanol prior to embedding in paraffin. The liver was sectioned into 5 μm thick sections using a rotary microtome (HistoCore BIOCUT, Leica Biosystems, Germany) and mounted onto glass slides, followed by dewaxing and rehydration steps. The specimens were stained with hematoxylin and eosin (H&E). They were then dehydrated and observed under an Olympus BX53 microscope (Olympus, Tokyo, Japan). At least six images per sample were acquired at 40x magnification.

### Microbiota analysis by 16S rRNA gene sequencing

Cecal content samples were used for analysis; equal amounts of cecal samples from two mice were pooled together, resulting in four pooled samples per group. Bacterial DNA extraction, 16S ribosomal RNA gene sequencing, and all bioinformatic analyses were performed by the Bioengineering Lab. Co., Ltd. (Kanagawa, Japan). DNA was extracted using Lab-Aid824s DNA extraction kit (Zeesan Biotech), and DNA concentrations were measured using Synergy LX (Bio-Tek) and QuantiFluor dsDNA System (Promega) following the manufacturer’s standard protocols. The first PCR was performed using the primers 341f_MIX (F) (5′-ACACTCTTTCCCTACACG ACGCTCTTCCGATCT- NNNNN-CCTA CGGGNGGCWGCAG-3′) and 805r_MIX (R) (5′-GTGACTGGAGTTCAGACGTGTGC TCTTCCGATCT-NNNNNGACTACHVGGGTATC TAATCC-3′). The second PCR was performed using primers F (5′-AATGATACGGCGACCA CCGAGATCTACAC-Index2-ACACTCTTTCCCTACACGACGC-3′) and R (5′-CAAGCA GAAGACGGCAT ACGAGAT-Index1-GTGACTGGAGTTCAGACGTGTG-3′). Barcoded V3–V4 PCR amplicons were sequenced using an Illumina MiSeq next-generation sequencer at 2 × 300 bp. Sequencing data were processed and analyzed using Quantitative Insights into Microbial Ecology (QIIME2 (2022.8)). The high-quality sequences were clustered into operational taxonomic units (OTUs) with 97% sequence similarity, and OTUs were assigned to the Greengene database.

### Statistical analysis

All data were analyzed using Statistical Package for Social Science (SPSS) software version 26 or GraphPad Prism 10 (GraphPad Software, CA, USA). To measure the effects of the RBW–RBO oleogel in the food matrix model on the gut function of HFD-fed mice, an analysis of variance was performed with a significance set at *P* < 0.05. Significant differences among mean values were determined using Duncan’s Multiple Range Test. Cecal microbiota data were analyzed using the Kruskal–Wallis one-way ANOVA. Results are expressed as means ± standard deviation (SD).

## Supplementary information

Supplementary data

## Data Availability

All other data is available from the authors upon request.
