# Supplementary material for: Effects of RBX oleogel and heat–moisture-treated rice flour in food matrices on digestibility and microbiota
Source: NPJ Sci Food. 2025 Apr 22;9:54. doi: 10.1038/s41538-025-00418-7 (PMC12015495; doi:10.1038/s41538-025-00418-7)
Supplement: Supplementary file 1 — Supplementary data [file 41538_2025_418_MOESM1_ESM.pdf]

Supplementary Tables

Table S1 The composition of experimental high-fat diets

Constituents	Experimental diets (%, w/w dry weight)					
	Individual components				Food matrix	
	Beef tallow		Oleogel			
	Raw	Cooked	Raw	Cooked	Cooked	
	HMTF+B	HMTG+B	HMTF+O	HMTG+O	HMTGO	NGO
Beef tallow (B)	30.0	30.0	-	-	-	-
RBW-RBO oleogel (O)	-	-	30.0	30.0	30.0	30.0
HMT rice flour (HMTF) [c]	23.0	-	23.0	-	-	-
HMT rice flour gel (HMTG) [c]	-	23.0	-	23.0	23.0	-
Rice flour gel (NG) [c]	-	-	-	-	-	23.0
Casein [a]	18.4	18.4	18.4	18.4	18.4	18.4
L-cystine	0.3	0.3	0.3	0.3	0.3	0.3
Vitamin mixture [b]	1.0	1.0	1.0	1.0	1.0	1.0
Mineral mixture [b]	3.5	3.5	3.5	3.5	3.5	3.5
Cellulose	5.0	5.0	5.0	5.0	5.0	5.0
Sucrose	20.0	20.0	20.0	20.0	20.0	20.0
Water (in dried gel)	-	29.0	-	29.0	29.0	29.0
Total Solid	101.2	101.2	101.2	101.2	101.2	101.2
Total Food	101.2	130.2	101.2	130.2	130.2	130.2
Excess water	-	29.0	-	29.0	29.0	29.0

[a]20% w/w dry basis of total protein was calculated from casein (87% w/w net protein content) and native rice flour.
[b]American Institute for Nutrition-93.
[c]20.2%w/w dry basis of starch was calculated from native rice flour.

Table S2 Resistant starch content

Treatment	Resistant starch content (g/100g)**
NF	1.59 ± 0.19^{e}
HMTF	4.10 ± 0.05^{a}
HMTG	3.63 ± 0.01^{b}
HMTGO_10*	2.69 ± 0.01^{c}
HMTGO_20*	1.93 ± 0.01^{d}

All data were reported as means ± standard deviations ($n = 2$). Values with different superscript letters in the same column are significantly different ($P < 0.05$).

NF: native flour; HMTF: heat-moisture treated flour; HMTG: heat-moisture treated flour gel; HMTGO_10: the HMT starch-oleogel food matrix with 10% w/w oleogel, prepared by mixing the oleogel (10% w/w) with HMTF, followed by gelatinization; HMTGO_20: the HMT starch-oleogel food matrix with 20% w/w oleogel, prepared by mixing the oleogel (20% w/w) with HMTF, followed by gelatinization.

*HMT rice flour gel with RBW oleogel addition: The 20% w/w HMT rice flour gel with various RBW oleogel concentrations (0, 10, and 20% w/w based on rice flour weight) was prepared as follows. The RBW oleogel, HMT rice flour (HMTF), and distilled water were weighed. Then, the mixture was mixed by magnetic stirrer at 25°C for 10 min. Then, the mixture was heated and stirred on a hot plate stirrer until the paste temperature reached 60°C. Consequently, the pastes were poured onto the aluminum trays and steamed for 20 min before cooling at 25°C for 3 h.

**Resistant starch (RS) content of all samples was quantified using the 2, (K-RSTAR, Megazyme Bray, Co. Wicklow, Ireland) by following the AOAC Official Method 2002.02.

Table S3 Pasting properties of native and HMT rice flour with oleogel.

Parameters	Treatments**		
	**HMTF**	**HMTGO_10**	**HMTGO_20**
Pasting Temperature (°C)	81.33 ± 0.88	81.35 ± 0.85	80.53 ± 0.88
Peak Viscosity (cP)	4582 ± 30	4622 ± 142	4775 ± 29
Trough Viscosity (cP)	3835 ± 4	3600 ± 295	3707 ± 80
Breakdown Viscosity (cP)	747 ± 35	1022 ± 153	1068 ± 109
Final Viscosity (cP)	8071 ± 78^{c}	8630 ± 216^{b}	9376 ± 187^{a}
Setback* (cP)	4236 ± 82^{c}	5030 ± 79^{b}	5669 ± 267^{a}

All data were reported as means ± standard deviations (n = 2). Values with different superscript letters in the same row are significantly different (P < 0.05).

HMF: heat-moisture treated flour; HMTGO_10: the HMT starch-oleogel food matrix with 10% w/w oleogel, prepared by mixing the oleogel (10% w/w) with HMTF, followed by gelatinization; HMTGO_20: the HMT starch-oleogel food matrix with 20% w/w oleogel, prepared by mixing the oleogel (20% w/w) with HMTF, followed by gelatinization.

*Setback (Setback from Trough).

**The pasting properties of HMT rice flour incorporated with oleogel were determined by Rapid Visco Analyser (RVA) model 3D (Newport Scientific, Warriewood, Australia). The RBW oleogel was weighed to have final concentration of 0, 10, and 20% w/w flour basis in the aluminum RVA sample canister. The sample was weighed (3 g dry basis) directly into the aluminum RVA sample canister, and 25 mL distilled water was added and mixed by RVA paddles. The samples were dispersed at 960 r/min for 10 s, and then the speed was reduced to 160 rpm throughout the test. A programmed heating and cooling cycle were used where the samples were held at 50°C for 2 min, heated to 95°C in 7.5 min, held at 95°C for 5 min before cooling to 50°C in 7.5 min, and holding at 50°C for 5 min. Pasting temperature, peak, trough, breakdown, final, and setback viscosity were recorded. All samples were done in two replications.

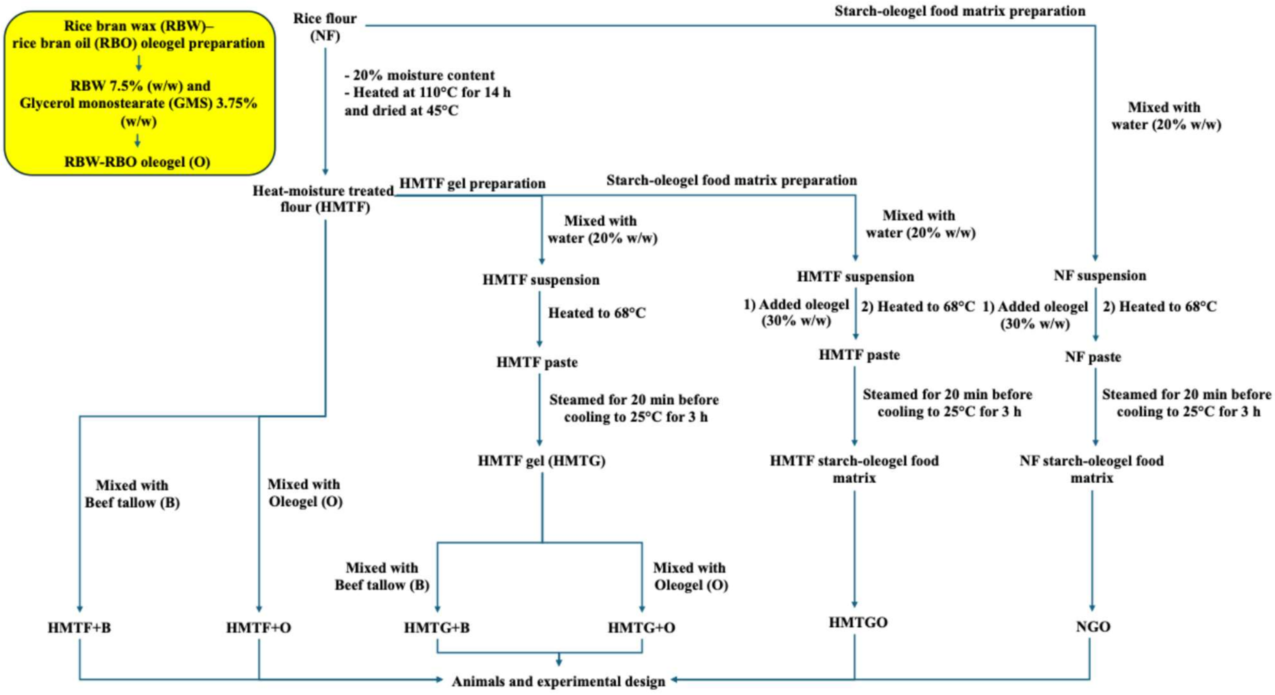

Fig. S1 A flow chart of sample preparation.

Supplementary Figures

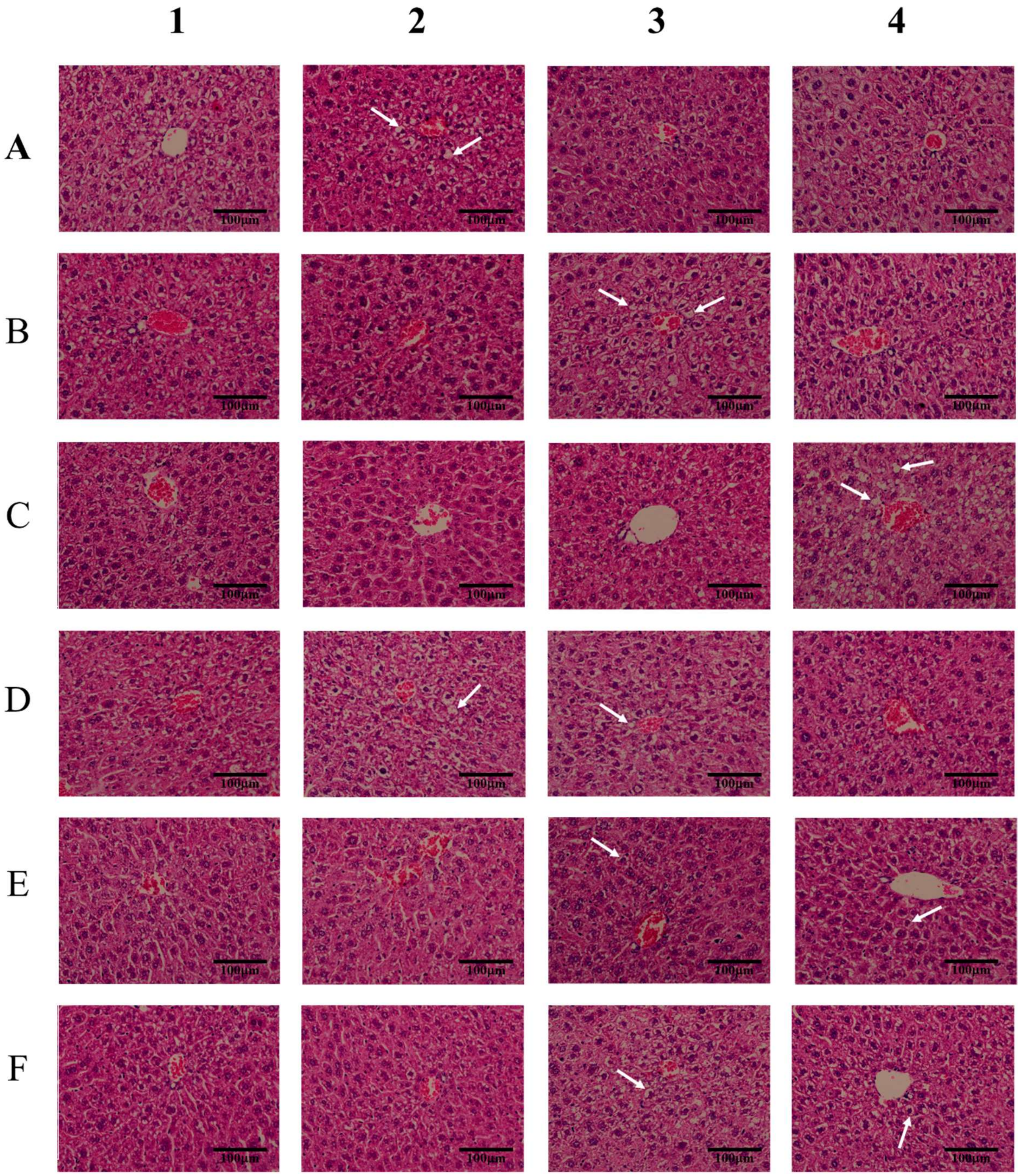

Fig. S2 The effect of RBW-RBO oleogel in the food matrix with HMT starch (in form of flour) on lipid accumulation in the liver. The liver sections stained with H&E (40X) from 4 mice/group. Arrows indicate fat cells. A: HMTF+B; B: HMTG+B; C: HMTF+O; D: HMTG+O; E: HMTGO; F: NGO

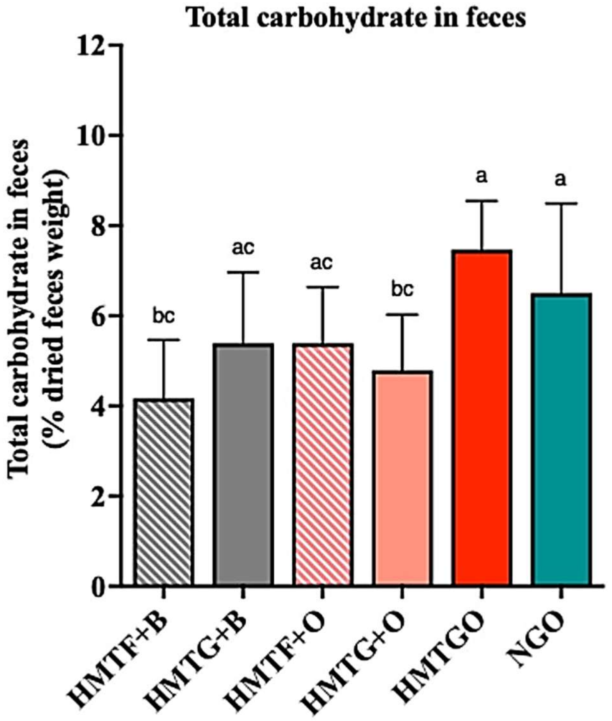

Fig. S3 Effects of individual components and food matrices on fecal carbohydrate excretion. The results are expressed as the means ± SD (n = 7–8 mice/group). One-way ANOVA followed by Tukey's multiple comparisons test was used to test significances between groups. All statistical analyses were done by using GraphPad Prism 10 (GraphPad Software, CA, USA). Different letters above the bars indicate significantly difference ($P < 0.05$). B: beef tallow, O: oleogel, HMTF: heat-moisture-treated flour, HMTG: HMTF gel, HMTGO: the HMT starch-oleogel food matrix, NGO: a native starch-oleogel food matrix.

The amount of total carbohydrate in fecal samples was determined by a phenol–sulfuric acid method (Dubois et al., 1956; Deans et al., 2018; Kokubo et al., 2022). Glucose was used as a standard and the measured value quantified as glucose equivalent. Briefly, dried feces (20 mg each) were mixed in MilliQ (10 mL), vortexed, and sonicated for 15 min. After centrifugation at 7000 g for 15 min at room temperature, each supernatant was diluted in MilliQ at a ratio of 1:1. After that, these diluted supernatants (0.25 mL) were mixed in 5% phenol solution (0.15 mL) and vortexed. Then, sulfuric acid (0.75 mL) was added into the mixture, gently mixed, and incubated at room temperature for 45 min. After the incubation, the mixtures were measured by a spectrophotometer at 490 nm. Glucose solutions (0.0025, 0.0125, 0.0375, and 0.075 mg/mL) were used for standard curve calculation.

DuBois, M., Gilles, K.A., Hamilton, J.K., Rebers, P.T. & Smith, F. Colorimetric method for determination of sugars and related substances. Analytical Chemistry. 1956; 28(3):350-6.

Deans, C.A., Sword, G.A., Lenhart, P.A., Burkness, E., Hutchison, W.D. & Behmer S.T. Quantifying plant soluble protein and digestible carbohydrate content, using corn (Zea mays) as an exemplar. Journal of Visualized Experiments: JoVE. 2018 (138).

Kokubo, E., Sonoki, H., Aizawa, K., Takagi, H., Takada, M., Ito, A., Nakazato, Y., Takeda, Y. & Miyaji, K. In Vivo Digestibility of Carbohydrate Rich in Isomaltomegalosaccharide Produced from Starch by Dextrin Dextranase. Journal of Applied Glycoscience. 2022; 69(3):57-63.

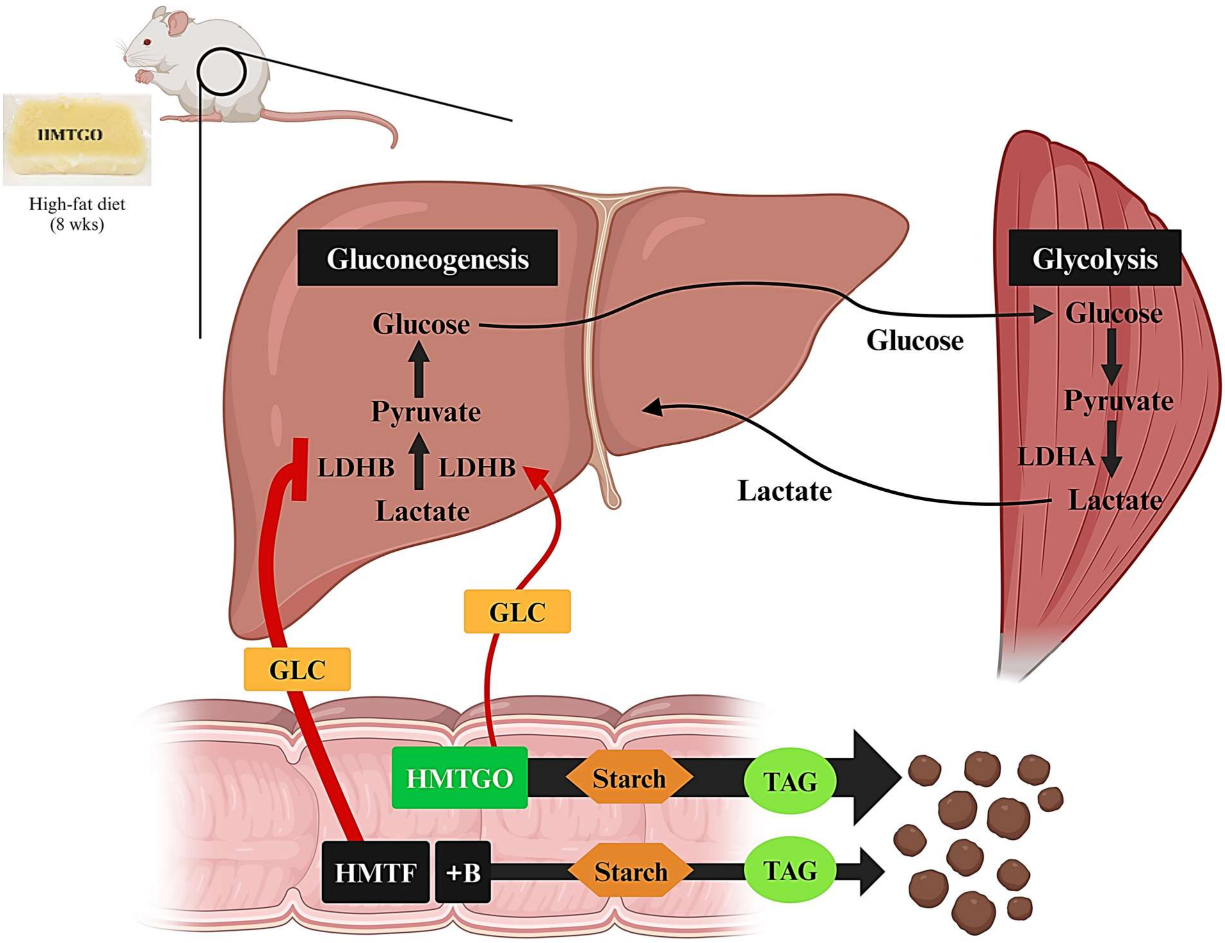

Fig. S4 LDH and blood glucose hypothesis.
LDH: Lactate dehydrogenase; GLC: Glucose; TAG: Triacylglycerol
This figure was created using Biorender.com.

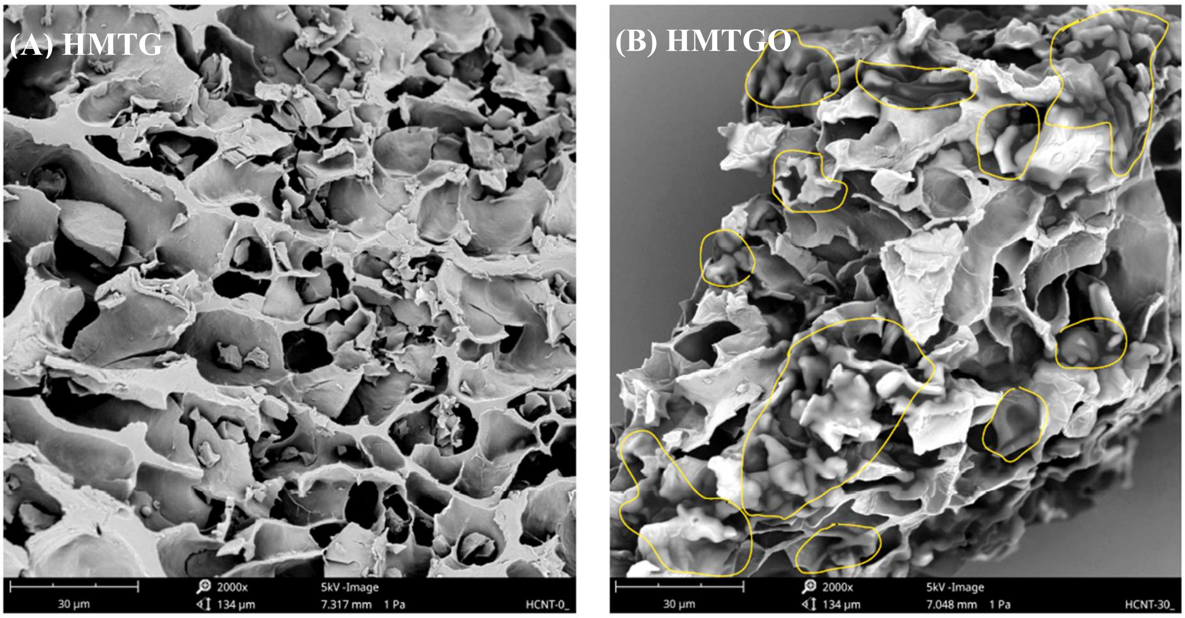

Fig. S5 Scanning electron micrographs of cooked HMTG (A) and HMTGO (B) samples. Both HMTG and HMTGO samples were cooked (gelatinized) before being subjected to SEM. In (B), the yellow circles highlight round-surfaced structures that we hypothesized to be lipid (oleogel) interacting with starch within the food matrix. Contents (% w/w dried weight) of HMT rice flour (23%) and oleogel (30%) were set the same with the experimental diet composition shown in Table S1. HMTG: HMTF gel, HMTGO: the HMT starch-oleogel food matrix. Scale bar = 30 μm.

A scanning electron microscope (Phenom ProX, Thermo Fisher Scientific, Netherlands) was used. The sample was spread on an SEM stub using double-sided adhesive tape and coated with gold. The microscope was operated at a voltage of 5 kV, with a magnification of 2000x.

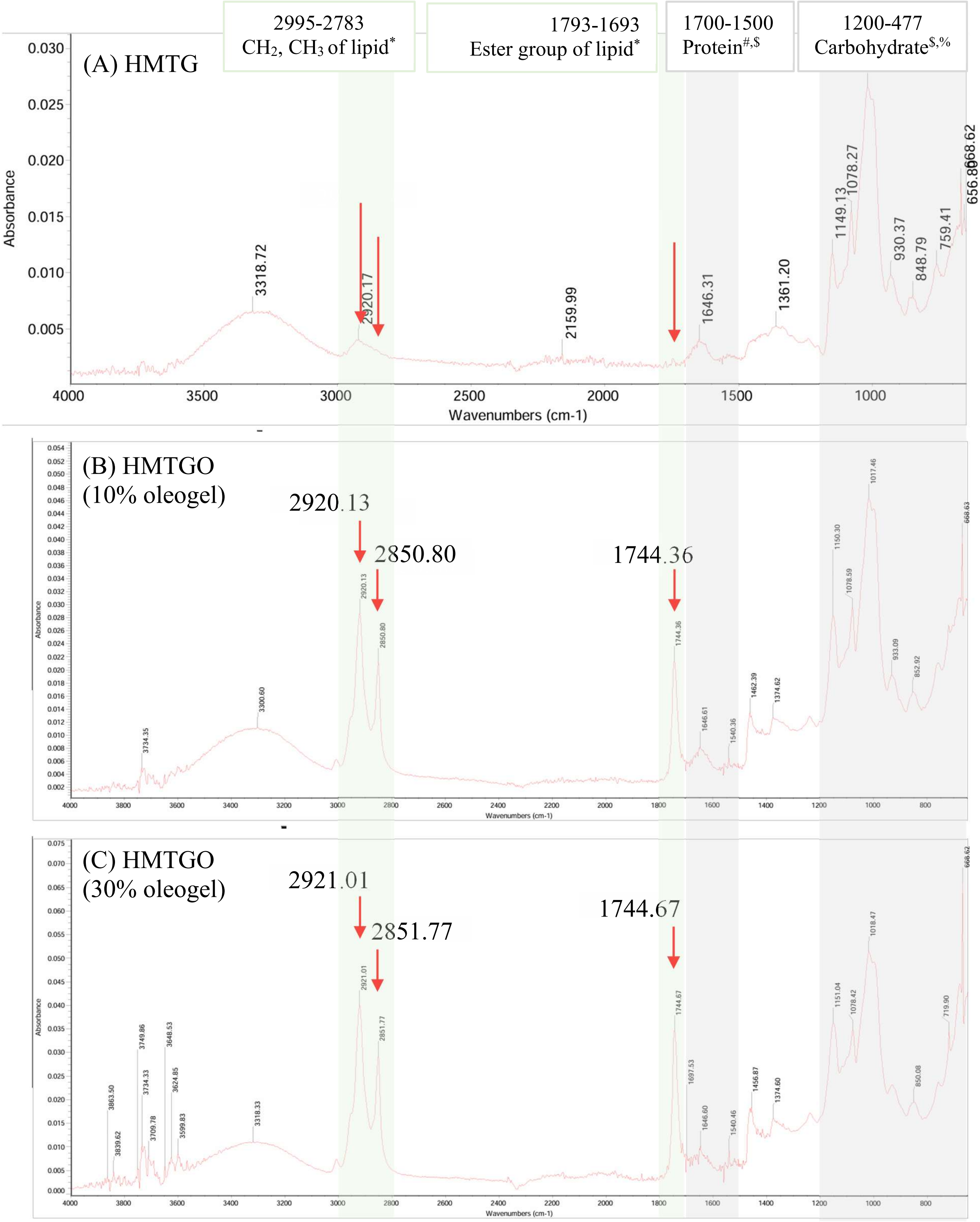

Fig. S6 FTIR spectra of HMTG (A), HMTGO (10% oleogel) (B), and HMTGO (30% oleogel) samples. HMTGO samples were cooked (gelatinized) before being subjected to the analysis. HMTF: heat-moisture-treated flour, HMTGO: the HMT starch-oleogel food matrix.

*Chen L, Tian Y, Sun B, Cai C, Ma R, Jin Z. Measurement and characterization of external oil in the fried waxy maize starch granules using ATR-FTIR and XRD. Food chemistry. 2018;242:131-8.

[#]Chen X, He X, Zhang B, Fu X, Li L, Huang Q. Structure, physicochemical and in vitro digestion properties of ternary blends containing swollen maize starch, maize oil and zein protein. Food Hydrocolloids. 2018;76:88-95.
[$]Ying D, Hlaing MM, Lerisson J, Pitts K, Cheng L, Sanguansri L, Augustin MA. Physical properties and FTIR analysis of rice-oat flour and maize-oat flour based extruded food products containing olive pomace. Food Research International. 2017;100:665-73.
[%]Flores-Morales A, Jiménez-Estrada M, Mora-Escobedo R. Determination of the structural changes by FT-IR, Raman, and CP/MAS 13C NMR spectroscopy on retrograded starch of maize tortillas. Carbohydrate Polymers. 2012;87(1):61-8.

The chemical structure of HMT rice flour gel (HMTG) and HMT rice flour gel with oleogel (HMTGO) were obtained using a Fourier Transform Infrared Spectrophotometer (FTIR) (Bruker Tensor 27, Germany) with an Attenuated Total Reflectance (ATR) accessory. Each measurement consisted of 64 scans in the range 400 to 4000 cm^{-1} at a resolution of 4 cm^{-1}. The deconvolution data was used Analyzer software (OriginPro 2018). The measurement was done in two replications.
